# Supplementary material for: Netrin-1 inhibits the attachment and internalization of Hepatitis B virus for hepatocyte infection
Source: PLoS Pathog. 2025 Dec 17;21(12):e1013776. doi: 10.1371/journal.ppat.1013776 (PMC12711026; doi:10.1371/journal.ppat.1013776)
Supplement: Attachment — Submitted filename: Response to Reviewers.docx [file ppat.1013776.s016.docx]

**Major Issues: Key Experiments Required for Acceptance**

**Reviewer #1:**

-------------------------------------------------------------------------------------------------------------------------

1. The authors propose that the extracellular secreted protein LIPG facilitates the attachment of HBV to the hepatocyte surface and promotes viral internalization. On the other hand, hepatocytes (PXB cells, Fig. 1B) also express the LIPG-binding protein Netrin-1, which inhibits the aforementioned process. Netrin-1 may also inhibit viral endocytosis by suppressing the interaction between NTCP and EGFR. Given this, how are these processes regulated temporally and spatially during natural HBV infection? Does the expression of Netrin-1 represent a host cell's antiviral defense mechanism following viral infection?

Based on the presented data, neither Huh7 nor HepG2-derived tumor cell lines express endogenous Netrin-1. Even in HBV-expressing HepG2.2.15 cells, Netrin-1 expression cannot be detected (Fig. 1D). Does this contradict the mRNA quantification data shown in Fig. 1B? Does it imply that even very low levels of Netrin-1 expression can strongly inhibit HBV infection? Given that Netrin-1 expression is barely detectable at the protein level, is the proposed mechanism of competitive protein binding involving Netrin-1 reasonable? The authors should also determine the endogenous expression levels of Netrin-1 in PXB cells.

-------------------------------------------------------------------------------------------------------------------------

We appreciate Reviewer 1 for the thoughtful and detailed comments. We agree that the regulation and physiological relevance of Netrin-1 expression during HBV infection are important issues. To answer the concerns of Reviewer 1, we evaluated Netrin-1 expression over the period of HBV infection in PXB and HepG2-derived cells. We detected appropriate levels of Netrin-1 in the cell lysate and culture supernatant of PXB cells by western blotting and ELISA, respectively, and the results are now shown in Supplemental Figure S4. Serum Netrin-1 is utilized as a biomarker of various diseases. Importantly, the concentrations of Netrin-1 in the culture supernatant of PXB cells were 30–90 pg/mL and they were comparable to those in normal human serum (130 pg/mL). Therefore, secreted Netrin-1 from normal hepatocytes potentially possesses biological functions. To obtain concrete evidence for the significance of secreted Netrin-1, we performed Transwell experiments, and the results are presented in Figure 1Q–T and Supplemental Figure S2. We showed that secreted Netrin-1 was transferred to non-expressing cells and inhibited HBV entry.

Unlike normal hepatocytes, hepatoma-derived cells expressed low levels of Netrin-1. We do not yet know the reason for this, as Netrin-1 is expressed at higher levels in neural tumor, lung cancer, breast cancer, kidney cancer, etc. Among HBV permissive hepatoma cells, we found HepG2-NTCPsec+ cells expressed fairly detectable levels of Netrin-1 by western blotting, although the concentrations in the cell supernatants were negligible. Interestingly, we found that Netrin-1 expression was reduced by HBV infection in both PXB and HepG2-NTCPsec+ cells. On the other hand, the expression of LIPG was upregulated, especially in the late phase of HBV infection of PXB cells (Supplemental Figure S4). Although the mechanisms for the reduction of Netrin-1 expression by HBV infection should be explored further, the results have potential implications for the functional role of Netrin-1 in HBV infection.

We added the following text regarding the analysis of Netrin-1 and LIPG expression:

“We evaluated the expression of LIPG and Netrin-1 over the course of HBV infection in PXB cells and HepG2 derived HBV permissive cells…………………….its general expression was gradually reduced over the culture period.” in the Results, page 6, lines 176–191.

“Netrin‑1 is a secreted laminin-related protein and plays a pivotal role in neural development and guiding axons…………. If Netrin-1 has different effects on HCV and HBV, in the case of HCV/HBV coinfection, the increased expression of Netrin-1 induced by HCV should return to normal by the eradication of HCV, which could potentially trigger HBV reactivation, as occasionally observed in direct-acting antiviral treatment” in the Discussion, page 17-18, lines 553–580.

“ELISA

The concentrations of Netrin-1 and LIPG in cell culture supernatants were measured using ELISA kits: CSB-E11899h (Cusabio, Wuhan, China) for Netrin-1 and MBS702323 (MyBioSource, San Diego, CA, USA) for LIPG, following the manufacturers’ instructions. Sample concentrations were calculated using the online Gaindata ELISA analysis tool.” in the Materials and Methods, page 23, lines 773–776.

We added the following descriptions of the Transwell experiments:

“Netrin-1 is a secreted protein and interacts with HSPGs on the cell surface………..…. Moreover, recombinant Netrin-1, freshly added to the culture medium, also inhibited HBV attachment and internalization in an approximately dose-dependent fashion (Supplemental Figure S3)” in the Results, pages 5–6, lines 159–175.

“Despite the appropriate expression of Netrin-1 in PXB cells………… Thus, the results clearly show that Netrin-1 regulates HBV entry in a *trans*-acting manner.” in the Discussion, page 15, lines 478–485.

“Transwell Co-culture Assay

Dox-inducible Netrin-1-overexpressing HepG2-NTCP-YFP cells were seeded……….. membrane fractions from the lower chamber cells, were prepared and analyzed to detect Netrin-1-HA expression levels.” in the Materials and Methods, pages 24, lines 799–812.

-------------------------------------------------------------------------------------------------------------------------

2. Fig. 2F: LIPG appears to be a highly sticky protein, interacting with various functional domains of Netrin-1. Is there a common structural basis for these interactions? Particularly noteworthy is that LIPG also binds to the L protein of HBV (Fig. 4B, C). Could additional experiments be designed, beyond co-immunoprecipitation (Co-IP) assays in cells, to further demonstrate direct interactions between purified proteins? Additionally, are all the proteins shown in Fig. 2G secreted outside the cell, thereby enabling their role in inhibiting viral attachment?

-------------------------------------------------------------------------------------------------------------------------

As Reviewer 1 pointed out, LIPG appears to be a highly sticky protein. Unfortunately, we have not yet identified the common structure in Netrin-1 for binding to LIPG. As Reviewer 1 mentioned, we think that the interaction between LIPG and LHBs (Figure 4A–D) is important. To explore the direct interactions between the short peptide LIPG-CTD-HBS (amino acids 331–340) and LHBs, we biotinylated LIPG-CTD-HBS. A pull-down assay of biotinylated protein-protein interactions showed LIPG-CTD-HBS bound directly to LHBs and this binding was competed out by recombinant Netrin-1 protein. These results are shown in Figure 4D.

We added the following descriptions:

“To demonstrate the direct interaction of LHBs and LIPG-CTD HBS, a biotinylated peptide of LIPG-CTD HBS………… These results indicate that Netrin-1 can compete with the direct interaction of LHBs and LIPG-CTD HBS.” in the Results, page 9-10, lines 293–301.

“Pull-down assay for biotinylated protein:protein interaction

A Pierce Pull-Down Biotinylated Protein:Protein Interaction Kit (21115; Thermo Fisher Scientific) was used……………. Detection was carried out using an anti-His tag antibody (for Netrin-1-His) and an anti-HBsAg antibody for large HBsAg, as appropriate.” in the Materials and Methods, page 23, lines 785–797.

To address Reviewer 1’s concern regarding Figure 2G, we evaluated the expression of Netrin-1 and its related subdomains in whole cell lysate, cell membrane, and culture supernatant.

We added the following descriptions:

“Western blotting analysis confirmed that all domains were detected and comparably expressed in both the whole cell lysate and membrane fraction…….. domains V and C, but not domain VI, suppressed HBV attachment to a similar extent as wild type Netrin-1 did (Figure 2H).” in the Results, page 8, lines 245–249.

“In this study, we performed functional analysis of individual domains (VI, V and C)………. The truncated conformational structures of these proteins might promote their oligomerization or aggregation.” in the Discussion, page 15-16, lines 502–508.

-------------------------------------------------------------------------------------------------------------------------

3. In my view, the experimental design in Fig. 7 is highly problematic, and is the aspect of this study that concerns me the most. The preceding logic is that Netrin-1 inhibits HBV infection of hepatocytes through various competitive protein-protein interactions on the cell surface. However, the construct designed in Fig. 7A surprisingly introduces an endosomal cleavage site, a fusogenic peptide, and an NLS peptide. What are their functions? Are they still intended to inhibit viral entry at the cell surface? Additionally, using PBS as a control in the in vivo experiments is clearly unreasonable. The authors should consider whether ASGR monoclonal antibodies also affect HBV infection of hepatocytes. Moreover, the naming of the LIPG peptide is also very confusing.

-------------------------------------------------------------------------------------------------------------------------

Reviewer 1 had questions about why we added the fusogenic peptide and NLS to Netrin-1-related peptides, because the HBV entry process is an event that occurs at the cell surface. Reviewer 1 is reasonable; however, we consider that the HBV entry process is not limited to the cell surface, but is closely related to post-cell-surface events, i.e., trafficking to the nucleus and cccDNA synthesis and repair. EGFR has been reported to be transferred to the nucleus by escaping from endosomes and avoiding lysosomal degradation (Clinical Cancer Research. 2009. pp. 6484–6489. doi:10.1158/1078-0432.CCR-08-2813). Nuclear EGFR binds to DNA protein kinases, participating in double-stranded DNA repair (Cancer Res. 2011;71: 1103–1114. doi:10.1158/0008-5472.CAN-10-2384). Crosstalk between DNA protein kinases and single-stranded DNA repair molecules, such as ATR, has been reported (Mol Cell. 2015;59: 603–614. doi:10.1016/j.molcel.2015.06.026). ATR is essential for HBV cccDNA synthesis (mBio 2020;11: e03423-19. doi:10.1128/mBio.03423-19). In addition, EGFR is associated with proliferating cell nuclear antigen, which is also involved in cccDNA synthesis (Nucleic Acids Res. 2024;52: 13057–13072. doi:10.1093/nar/gkae938).

The combination of a fusogenic peptide and NLS is an effective system for peptide transfer to the cytoplasm and then to the nucleus. It enables peptides to escape from endosomes by avoiding lysosomal degradation and facilitates their nuclear transfer. This system works well, as we reported previously (J Biol Chem. 2022 Jul;298(7):102097. doi: 10.1016/j.jbc). Although we did not obtain direct evidence, we assume that the Netrin-1-related peptide inhibits EGFR activation. Therefore, we retained this system to target the cell surface and nucleus. We apologize that the naming of the peptide is confusing; however, the optimization of codon sequences to yield a stable amount of peptide required multiple steps. The resultant final peptide, 10ML-B28, consists of an anti-ASGR antibody (ASGR3-10M), Netrin-1-related peptide (LIPH4-LNTNBS) followed by an NLS and S39 (fusogenic peptide). We assume that the anti-ASGR antibody concentrates 10ML-B28 on hepatocytes and inhibits HBV entry. Then, 10ML-B28 is endocytosed in endosomes and released from endosomes after avoiding lysosomal degradation, then transferred to the nucleus. Nuclear 10ML-B28 is expected to inhibit EGFR activation.

As Reviewer 1 pointed out, PBS as a control in the *in vivo* experiments may not be reasonable. Unfortunately, we have not yet identified an appropriate control peptide. However, we are now attempting to develop such a peptide. As random sequence-based peptides occasionally show non-specific biological activity depending on the experimental system, multiple cellular and mouse experiments should be performed to choose an appropriate control peptide. Nevertheless, we validated the anti-ASGR antibody (ASGR3-10M) for HBV entry as the control and the results are shown in Supplemental Figure S12. ASGR3-10M had no significant anti-HBV activity (from 10–100 nM).

We added the following description:

“The ASGR3-10M antibody is expected to concentrate these peptides on the cell surface; however, it did not have a significant effect on HBV infection (Supplemental Figure S12).” in the Results, pages 14, lines 438–440.

**Reviewer #2:**

-------------------------------------------------------------------------------------------------------------------------

1. Concerns about relevance to natural infection: A more fundamental concern lies in the conceptual framing of the hypothesis. Even if we assume that the authors' experimental observations are accurate, it is difficult to reconcile the proposed mechanism with the known biology of viral infection. The central hypothesis posits that Netrin-1 inhibits HBV internalization and attachment, which are events that occur extracellularly or at the cell surface. However, most of the experiments described in the manuscript focus on intracellular interactions—specifically, protein-protein interactions, displacement assays, and competition experiments within the cell. More importantly, Netrin-1 is a secretory protein, as the authors themselves acknowledge in the manuscript. In light of this, the authors need to provide a strong justification for why the intracellular interactions among LIPG, LHBs, and Netrin-1 are expected to yield biologically relevant insights.

This raises an important question: how do these intracellular events mechanistically relate to the inhibition of viral entry, which is proposed to occur at the cell surface? Before delving further into the technical details of the experiments, the authors need to more clearly explain how their hypothesis and current data connect to the natural course of viral infection. Without this clarification, the physiological relevance of the findings remains uncertain.

-------------------------------------------------------------------------------------------------------------------------

-------------------------------------------------------------------------------------------------------------------------

2. The proposed model should be defined more clearly: The authors should clearly distinguish whether the effects of ectopic expression of Netrin-1 (and its various variants) are exclusively cis-acting or if they can also function in trans. Given that Netrin-1 is a secreted protein and the overall hypothesis appears to operate in the extracellular environment (as depicted in Figures 2A, 4J, and 5H), it is important to experimentally address this distinction. One way to test this would be to use cells overexpressing Netrin-1 and assess a relevant effect, such as viral attachment or internalization (that authors have done it already). These Netrin-1 overexpressing cells could be co-cultured with control cells (which do not overexpress Netrin-1) in a transwell system. If the authors’ hypothesis is correct and Netrin-1 acts in trans, the control cells should partially adopt the phenotype observed in Netrin-1 overexpressing cells due to secreted signaling molecules. Conversely, if the effect is strictly cis-acting, the control cells would remain unaffected (for example no effect on viral internalization), and the phenotype would only be observed in the cells directly overexpressing Netrin-1. This experiment would provide critical insight into the mechanism of Netrin-1 action and help clarify whether its signaling is cell-autonomous or involves intercellular communication.

-------------------------------------------------------------------------------------------------------------------------

Reviewer 2 has provided us with very thoughtful and constructive comments. We appreciate the fruitful virological and scientific input. Reviewer 2 also suggested additional experiments. We reply to Comments 1 and 2 together.

As Reviewer 2 suggested, we performed Transwell experiments. The results are presented in Figure 1Q–T and Supplemental Figure S2. We showed that secreted Netrin-1 was transferred to non-expressing cells and inhibited HBV entry.

We added descriptions of the Transwell experiments as follows:

“Netrin-1 is a secreted protein and interacts with HSPGs on the cell surface………..…. Moreover, recombinant Netrin-1, freshly added to the culture medium, also inhibited HBV attachment and internalization in an approximately dose-dependent fashion (Supplemental Figure S3)” in the Results, pages 5–6, lines 159–175.

“Despite the appropriate expression of Netrin-1 in PXB cells………… Thus, the results clearly show that Netrin-1 regulates HBV entry in a *trans*-acting manner.” in the Discussion, page 15, lines 478–485.

“Transwell Co-culture Assay

Dox-inducible Netrin-1-overexpressing HepG2-NTCP-YFP cells were seeded……….. membrane fractions from the lower chamber cells, were prepared and analyzed to detect Netrin-1-HA expression levels.” in the Materials and Methods, pages 24, lines 799–812.

As Reviewer 2 pointed out, our protein-protein interaction analyses were mainly performed by cell transfection assays. All constructs used in this study (various truncations of Netrin-1 and LIPG) were cloned from the original signal sequence as described in the Materials and Methods, and we expected that the expressed proteins would be secreted and attach to the cell membrane. We assume that our results reflect the proteins at the cell membrane or in the medium adjacent to the cell membrane (Figure 2G). However, the possibility remains that our data may reflect intracellular proteins, although Transwell experiments clearly showed that secreted Netrin-1 inhibited HBV infection in a *trans*-acting manner.

We included this as a limitation of the study as follows:

“Another limitation of this study is that our protein-protein interaction analyses were mainly performed using a cell transfection assay. Netrin-1 and LIPG are secreted into the culture medium and attach to HSPGs on the cell membrane, and the levels of these proteins in the medium and cell membrane are similar.…………………………. Further analysis should be carried out to examine whether our data from the culture medium are valid by collecting secreted proteins individually.” in the Discussion, page 18, lines 587–599.

-------------------------------------------------------------------------------------------------------------------------

3. Lack of details and experimental clarity: One of the weaker aspects of the manuscript is the figure legends, which lack sufficient details and consistency. Furthermore, there are cases where the claims made in the text are not convincingly supported by the data presented in the figures. There are also multiple instances where the authors could have used simpler, more straightforward experimental strategies to validate their conclusions. Instead, they have chosen more complex approaches, which may not enhance the scientific value of the study and, in some cases, obscure the interpretation of the data.

-------------------------------------------------------------------------------------------------------------------------

In response to Reviewer 2’s concerns regarding the lack of detail and clarity in the figure legends, we have thoroughly revised all legends to ensure that each contains sufficient methodological information, experimental conditions (e.g., concentrations, incubation times, and temperatures), and labeling details to enhance clarity and reproducibility. Regarding the consistency between claims in the text and the data presented in the figures, we carefully re-evaluated the relevant sections and made necessary modifications to improve consistency.

**Reviewer #3: No specific comment.**

**Minor Issues: Editorial and Data Presentation Modifications**

**Reviewer #1**

-------------------------------------------------------------------------------------------------------------------------

1. Fig. 1D: cccDNA serves as the template for viral replication and typically exists in low copy numbers within cells. From a stoichiometric perspective, the intensity of the cccDNA band should not appear similar to that of the rcDNA (relaxed circular DNA) band. Additionally, why were the bands for the replication intermediates, such as Dsl DNA (double-stranded linear DNA) and SS DNA (single-stranded DNA), not observed in the figure?

-------------------------------------------------------------------------------------------------------------------------

As Reviewer 1 pointed out, the original Figure 1D did not show Dsl DNA and SS DNA.

We repeated the Southern blotting experiment and now present a revised Figure 1D. We showed clear Dsl DNA and SS DNA. Reviewer 1 claimed there was a larger amount of cccDNA than rcDNA in the original Figure 1D. However, repeated Southern blots also showed a larger amount of cccDNA than rcDNA.

When we looked back at our previous paper, Southern blotting of PXB cells showed similar patterns as in the present study, i.e., a larger amount of cccDNA than rcDNA, while there was a lower amount of cccDNA than rcDNA in HepG2-NTCP-C4 cells (Cell Mol Gastroenterol Hepatol 2023;15(3):533-558).

Cell Mol Gastroenterol Hepatol 2023;15(3):533-558.

**PXB cells**

Cell Mol Gastroenterol Hepatol 2023;15(3):533-558.

**HepG2-NTCP-C4**

We speculate this might be due to the different initial infectivity of PXB and HepG2-NTCP-C4 cells. PXB cells showed much higher infectivity than HepG2-NTCP-C4 cells, thus a larger amount of cccDNA was generated. It might also be influenced by transcriptional activity and releasing activity of infectious viral particles. These may be interesting points about the different replication machineries in PXB and HepG2-NTCP-C4 cells.

-------------------------------------------------------------------------------------------------------------------------

2. Lines 162–167: The description of HSPG-dependent and HSPG-independent HBV attachment in this section is confusing. The results in Fig. 2C actually suggest that the anti-HBV mechanism of Netrin-1 is downstream of Heparanase activity, meaning it is HSPG-dependent.

-------------------------------------------------------------------------------------------------------------------------

As Reviewer 1 pointed out, the description could be confusing. We modified the description as follows:

“In the process of entry into hepatocytes, HBV attaches to HSPGs on their surface with low affinity and then interacts with NTCP in a high-affinity manner[4]. In addition, HBV can attach to hepatocytes in an HSPG-independent manner as a preS1-probe was shown to interact directly with NTCP without any interference by heparin (Figure 2A)[6]. LIPG might stimulate HSPG-dependent and HSPG-independent HBV attachment, as reported previously[6].

To explore the role of Netrin-1 in HSPG-independent HBV attachment, we examined its effect on the attachment of the preS1-probe to the surface of HepG2-NTCP-YFP cells (Figure 2B). Interestingly, there was no decrease in preS1-probe attachment to the surface of Dox-induced Netrin-1-overexpressing cells (Figure 2B). These results suggest that Netrin-1 has no effect on HSPG-independent HBV attachment.” in the Results, pages 6–7, lines 194–204.

-------------------------------------------------------------------------------------------------------------------------

3. Fig. 5D: The image in Fig. 5D lacks statistical analysis. Additionally, why were the NTCP signal and preS1 probe signal not merged for analysis?

-------------------------------------------------------------------------------------------------------------------------

The images now shown in Figure 5C (originally Figure 5D) correspond to the quantification data in Figure 5B (originally Figure 5C), representing the 6-h time point. The quantification includes statistical analysis as indicated in the figure.

As Reviewer 1 commented, we have merged the NTCP and preS1 probe signals and presented the images in the revised Figure 5C.

-------------------------------------------------------------------------------------------------------------------------

4. Fig. 6D: EGF stimulation induces EGFR internalization, which is known to be partially degraded via the lysosomal pathway. However, this well-documented phenomenon does not appear to be reflected in the results shown in Fig. 6. Additionally, it is suggested to directly analyze the inhibitory effect of Netrin-1 on EGF-stimulated EGFR internalization using image data.

-------------------------------------------------------------------------------------------------------------------------

Thanks for the insightful comment. We acknowledge that EGFR internalization upon EGF stimulation is known to lead to partial degradation via the lysosomal pathway. However, since our experiment focused on assessing EGFR internalization rather than its degradation, we performed detection 15 minutes after EGF stimulation to capture early internalization events. This likely explains why lysosomal degradation was not clearly reflected in the images shown in Fig 6.

As Reviewer 1 suggested, we have now performed image-based quantification to assess directly the inhibitory effect of Netrin-1 on EGF-induced EGFR internalization. The results are presented in the revised Supplemental Figure S10.

We added the following description:

“In addition, EGF induced the internalization of the preS1-probe, and EGFR was clearly reduced by Dox-induced Netrin-1 overexpression (Supplemental Figure S9 and S10).” in the Results, page 16, lines 527–529.

**Reviewer #2:**

-------------------------------------------------------------------------------------------------------------------------

1. Why different scheme was adopted for Fig 1A and S1 Fig A (detection times were day 10 and day 17 respectively). Did authors check the effect of exogenous addition of rNetrin-1 (what was done in fig 1E) on HBV infection using PXB cells (like fig 1 C and D). If the central hypothesis is true, the authors should see some effect which will be opposite to what is reported in fig 1C and 1D.

-------------------------------------------------------------------------------------------------------------------------

We appreciate these important questions from Reviewer 2.

In Supplemental Figure S1A, the detection time point was set to day 17 because the experiment was designed as a screening assay to evaluate multiple candidate factors that might affect HBV infection. This longer incubation allowed us to capture cumulative effects across the infection process. According to these screening results, we focused on Netrin-1 and performed further experiments to assess its role in HBV entry. For this purpose, in Figure 1A, the detection time point was set to day 10, which is a more time saving and ideal point for the study of viral entry events.

Regarding your second point, we have previously examined the effect of exogenous recombinant Netrin-1 on HBV infection in PXB cells. These results were reported in our earlier publication (Ide et al., FEBS Letters, 2025, Figure 2B), which demonstrated a significant impact of recombinant Netrin-1 on HBV infection in this model.

-------------------------------------------------------------------------------------------------------------------------

2. For the figure 1 A and B, did authors check the protein depletion using western blot? What is the authors’ observation on endogenous expression of Netrin-1? Can authors provided western blot for cells they used to see how abundant this protein is? It will be very informative to know how much Netrin-1 is produced, retained within the cells, and secreted out.

-------------------------------------------------------------------------------------------------------------------------

To address Reviewer 2’s concerns, we evaluated Netrin-1 expression over the period of HBV infection in PXB and HepG2-derived cells. We detected appropriate levels of Netrin-1 in cell lysates and culture supernatants from PXB cells by western blotting and ELISA, respectively, and the results are shown in Supplemental Figure S4. Serum Netrin-1 is utilized as a biomarker of various diseases. Importantly, the concentrations of Netrin-1 in the supernatants of PXB cells were 30–90 pg/mL and they were comparable to those observed in normal human serum (130 pg/mL).

Unlike normal hepatocytes, hepatoma-derived cells expressed low levels of Netrin-1. Among HBV-permissive hepatoma cells, we found that HepG2-NTCPsec+ cells expressed detectable levels of Netrin-1 by western blotting, although the concentrations in the cell supernatants were negligible. Interestingly, we found that Netrin-1 expression was reduced by HBV infection in PXB and HepG2-NTCPsec+ cells. Conversely, LIPG expression was upregulated, especially in the late phase of HBV infection in PXB cells (Supplemental Figure S4). Although the mechanisms underlying the reduction of Netrin-1 expression by HBV infection should be explored further, these results have potential implications for the functional role of Netrin-1 in HBV infection.

We have added the following descriptions regarding the analysis of Netrin-1 and LIPG expression:

“We evaluated the expression of LIPG and Netrin-1 over the course of HBV infection in PXB cells and HepG2 derived HBV permissive cells…………………….its general expression was gradually reduced over the culture period.” in the Results, page 6, lines 176–191.

“Netrin‑1 is a secreted laminin-related protein and plays a pivotal role in neural development and guiding axons…………. If Netrin-1 has different effects on HCV and HBV, in the case of HCV/HBV coinfection, the increased expression of Netrin-1 induced by HCV should return to normal by the eradication of HCV, which could potentially trigger HBV reactivation, as occasionally observed in the direct-acting antiviral treatment” in the Discussion, page 17-18, lines 553–580.

“ELISA

The concentrations of Netrin-1 and LIPG in cell culture supernatants were measured…….. Sample concentrations were calculated using the online Gaindata ELISA analysis tool.” in the Materials and Methods, page 23, lines 773–777.

-------------------------------------------------------------------------------------------------------------------------

3. Which antibody was used in fig. 1F and 1j? Current labeling says “Netrin-1” and respective legend doesn’t provide any detials. If the Netrin-1 construct is tagged, such information shouldbe provided in the figure, if it was anti-Netrin-1 antibody then discuss why no band is visible in the control lane.

-------------------------------------------------------------------------------------------------------------------------

The Netrin-1 construct used in Figure 1F and 1J was indeed labeled with an HA tag. The immunoblotting in these panels was performed using an anti-HA antibody. We have revised the figure legends for Figure 1F and 1J to state that detection was carried out using an anti-HA antibody. In addition, we have updated the figure labels from “Netrin-1” to “Netrin-1 (α-HA)” to indicate clearly the construct and detection method used. These revisions should clarify the identity of the antibody used and the interpretation of the bands shown.

-------------------------------------------------------------------------------------------------------------------------

4. Control lane of fig 1M, in which authors have used anti-Netrin-1 antibody, shows that endogenous level of this protein is very low as hardly any band is detectable. This further substantiates the need to address the second half of the above comment #2.

-------------------------------------------------------------------------------------------------------------------------

In consideration of this point and the related Comment #2, we examined the endogenous expression levels of Netrin-1 in primary human hepatocytes (PXB cells) and HepG2-derived tumor cells (revised Supplemental Figure S4), as described in our response to Comment #2.

-------------------------------------------------------------------------------------------------------------------------

5. Line 196 and 198, the figure 1E should be figure 2E.

-------------------------------------------------------------------------------------------------------------------------

We have corrected the figure call out in lines 196 and 198 from “Figure 1E” to “Figure 2E” as suggested.

-------------------------------------------------------------------------------------------------------------------------

6. Figure 4I needs normalization control for membrane protein. Authors can choose any known protein that resides in membrane fraction and show it as a control to prove that membrane fractions were loaded equally.

-------------------------------------------------------------------------------------------------------------------------

In response to Reviewer 2’s suggestion, we have included Na⁺/K⁺-ATPase α1 (ATP1A1) as a normalization control for the membrane fraction in Figure 4J (originally Figure 4I). ATP1A1 is a well-established membrane-resident protein and is used widely as a loading control for membrane protein analysis. The graph showing LIPG and Netrin-1 intensity in the membrane fraction has been revised after normalization to ATP1A1.

-------------------------------------------------------------------------------------------------------------------------

7. Figure 5 needs some clarification. I am not sure if the experiment 5A is fully relevant to the hypothesis authors proposed here. The best answers will be provided by experiment when Netrin1 is exogenously added to these cells (and not ectopically expressed) and then see if Netrin affects 1068 p-EGFR level. If the authors believe the model proposed in fig. 5H, then exogenous addition of purified Netrin-1 should also affect EGFP 1068 phosphorylation or EGFR-NTCP interaction as shown in 5G. Fortunately, the authors seem to have access to purified rNetrin-1 protein that they have used in fig. 1 E and elsewhere.

-------------------------------------------------------------------------------------------------------------------------

Considering that the original Figure 5A may not be fully relevant to the hypothesis, we have removed it from the revised version. In accordance with this comment and the related Comment #8, we performed additional experiments using exogenously added recombinant Netrin-1. As shown in the newly added Figure 6E, treatment with recombinant Netrin-1 suppressed EGF-induced EGFR phosphorylation at Tyr1173 as well as the overall tyrosine phosphorylation of EGFR.

We added the following description:

“The reduction of EGFR phosphorylation (p-EGFR-1173 and p-EGFR-total) was also observed in recombinant Netrin-1 treated HepG2-NTCP-YFP cells (Figure 6E).” in the Results, page 12, lines 396–398.

-------------------------------------------------------------------------------------------------------------------------

8. Figure 6, it will further strengthen the hypothesis if authors can perform few of these experiments (wherever feasible) using exogenously added r-Netrin-1.

-------------------------------------------------------------------------------------------------------------------------

As part of our response to Comment #7, we performed additional experiments using exogenously added recombinant Netrin-1 and confirmed its ability to suppress EGF-induced EGFR phosphorylation (revised Figure 6E) as well as HBV entry (revised Supplemental Figure S3). We believe that this set of experiments should address Reviewer 2’s concern and further reinforce the proposed role of Netrin-1 in modulating EGFR signaling and viral entry.

We added the following description:

“Moreover, recombinant Netrin-1, freshly added to the culture medium, also inhibited HBV attachment and internalization in nearly dose dependent fashion (Supplemental Figure 3).” in the Results, page 6, lines 173–175.

-------------------------------------------------------------------------------------------------------------------------

9. More details should be provided for the in vivo treatment exp. How many micer in each group? What did the viremia look like prior to treatment during the 7 weeks? These are immunodeficient mice and should not mount any antiviral response so it is unclear about the ALT differences between the two groups. Why did the ALT levels went up over during treatment with PBS? Given the peptide treatment mainly targeted viral entry, one would expect the viremia to rebound to the control level in the treated mice after treatment. The authors should have monitored these mice after the treatment.

-------------------------------------------------------------------------------------------------------------------------

To address Reviewer 2’s thoughtful comments regarding the *in vivo* treatment experiment, we have revised the text and figure legend to provide the following clarifications:

1.	Animal numbers: The PBS-treated group consisted of three mice, and the peptide-treated group consisted of four mice. This information has now been included in the revised figure legend and the Materials and Methods.

2.	AST levels: We measured AST levels. As ALT assays have cross reactivity between mice and humans, AST levels directly reflect human hepatocyte derivatives. As noted by Reviewer 2, the observed difference in AST levels between the groups was unexpected. To address this, we re-measured the stored serum samples but there was still a trend that the AST levels were greater in the PBS group than in the treated group, although no significant difference in AST levels was observed between both groups (revised Figure 7F). We speculate that the reduction of HBV viral load may have prevented HBV-induced hepatocyte apoptosis. Further analysis should be performed to examine hepatocyte damage in the control and treated mice.

3.	Pre- and post-treatment viremia: In this study, we did not perform longitudinal measurements of viremia before and after peptide treatment, nor did we continue monitoring the mice after treatment cessation. We acknowledge that such data would provide valuable insights into the dynamics of viral rebound, especially considering the peptide’s mechanism targeting viral entry. We plan to incorporate extended viremia monitoring in future experiments to characterize better the treatment effects and viral kinetics.

-------------------------------------------------------------------------------------------------------------------------

10. The Discussion section requires substantial improvement. In its current form, it largely restates the results without providing deeper analysis or interpretation. The authors should incorporate additional relevant information to enhance this section. For example, the level of Netrin-1 mRNA in HBV-positive cohorts has been reported previously (see Plissonnier et al., PLoS Biology, 2016). Furthermore, more attention should be given to the nature of the current hypothesis. It would be valuable to clarify whether the proposed Netrin-1 signaling functions primarily in a cis-acting or trans-acting manner. Finally, the authors should acknowledge the limitations of their current model. What questions remain unanswered? What aspects should be addressed in future studies to advance understanding of this signaling pathway? Adding these elements will greatly strengthen the Discussion and provide clearer context for the study’s implications.

-------------------------------------------------------------------------------------------------------------------------

In response to Reviewer 2’s thoughtful and constructive comments regarding the Discussion, we have extensively revised this section to include deeper analysis and broader interpretation of our findings. As suggested, we clarified that Netrin-1 signaling functions primarily in a *trans*-acting manner, based on our co-culture experiments. This clarification also allowed us to explain better the temporal and spatial regulation of Netrin-1 during natural HBV infection, highlighting its potential role in modulating viral spread in a region- and stage-specific manner.

**Reviewer #3:**

-------------------------------------------------------------------------------------------------------------------------

1. For the experimental results presented in Fig. 1A to 1D, while shRNA#1 less efficiently reduced Netrin-1 mRNA in PXB cells (Fig. 1B), it more efficiently increased intracellular HBV DNA than shRNA#2. Why?

-------------------------------------------------------------------------------------------------------------------------

Thank you for pointing out this inconsistency. To address this, we repeated the experiment using freshly prepared samples and carefully standardized the experimental conditions, including shRNA transduction efficiency and timing of sample collection. Upon reanalysis, we found that the new data exhibited greater consistency across shRNA#1 and shRNA#2, with a clear correlation between Netrin-1 knockdown efficiency and intracellular HBV DNA levels. These updated results are now reflected in the revised version of Figure 1B–C, and we believe they more accurately represent the relationship between Netrin-1 suppression and HBV DNA in PXB cells.

-------------------------------------------------------------------------------------------------------------------------

2. Fig. S1. Lack of data to demonstrate the shRNA knockdown efficiency on their respective target RNA.

-------------------------------------------------------------------------------------------------------------------------

In response to this important comment, we have now included quantitative RT-PCR data demonstrating the knockdown efficiency of each shRNA on their respective target RNA. These results have been added to the revised Supplementary Figure S1, showing the reduction in mRNA levels in PXB cells transduced with each shRNA construct, relative to control. In addition, based on the knockdown efficiency results, we replaced the original six candidate factors with a new set of five factors that showed more robust silencing. We believe these additions address Reviewer 3’s concern and provide clearer rationale and validation for the gene knockdown approach used in our study.

-------------------------------------------------------------------------------------------------------------------------

3. For the experimental results presented in Fig. 1G, the concentration of recombinant Netrin-1 should be indicated in the Figure or figure legend.

-------------------------------------------------------------------------------------------------------------------------

The concentration of recombinant Netrin-1 used in the experiment shown in Figure 1H was 200 nM. This information has been included in the experimental design diagram in Figure 1E and added to the figure legend of Figure 1H to enhance clarity and ensure the reproducibility of the experimental conditions.

-------------------------------------------------------------------------------------------------------------------------

4. For the experiment presented in Fig. 2C, how long the cells were treated with Dox to induce Netrin-1 expression? This procedure should be included in the experimental schedule diagram on the left panel.

-------------------------------------------------------------------------------------------------------------------------

In the experiment shown in Figure 2C, the Dox-inducible Netrin-1-overexpressing HepG2-NTCP-YFP cells were treated with Dox for 72 h to induce Netrin-1 expression. We have revised the figure legend to include this information and also updated the experimental schedule diagram (left panel of Figure 2C) to indicate clearly the timing of Dox treatment. These changes help ensure a clearer understanding of the experimental conditions.

-------------------------------------------------------------------------------------------------------------------------

5. For the data presented in Fig. 2G and H, have you determined the subcellular localization of the three Netrin-1 fragments? Whether the failure of Netrin-1 VI fragment to inhibit HBV attachment is due to its inability to be transported onto the cell surface?

-------------------------------------------------------------------------------------------------------------------------

To clarify the subcellular localization of the three Netrin-1 fragments, we performed additional experiments assessing their expression in the culture supernatant and cell membrane fractions by western blotting. Our results showed that the VI fragment, like the other fragments, was detected in the culture supernatant and cell membrane fractions. Therefore, the failure of the VI fragment to inhibit HBV attachment is unlikely due to its inability to be transported to the cell surface. This suggests that other structural or functional properties of the VI fragment, rather than its subcellular localization, may account for its lack of an inhibitory effect on HBV attachment. These new data have been included in the revised Figure 2G and the manuscript has been updated accordingly.

We greatly appreciate the reviewers’ thoughtful comments on our manuscript. We have thoroughly revised the manuscript based on their comments and suggestions. We hope that the revised manuscript is now suitable for publication in *PLOS Pathogens*.

Sincerely yours,

Masao Honda, MD, PhD

Department of Clinical Laboratory Medicine,

Kanazawa University Graduate School of Medical Science,

Kodatsuno 5-11-80, Kanazawa, 920-0942, Japan

+81-76-265-2601

Department of Gastroenterology,

Graduate School of Medicine,

Kanazawa University, Takara-Machi 13-1, Kanazawa 920-8641, Japan

Tel: +81-76-265-2235; Fax: +81-76-234-4250

E-mail: mhonda@m-kanazawa.jp
