# Supplementary material for: Netrin-1 inhibits the attachment and internalization of Hepatitis B virus for hepatocyte infection
Source: PLoS Pathog. 2025 Dec 17;21(12):e1013776. doi: 10.1371/journal.ppat.1013776 (PMC12711026; doi:10.1371/journal.ppat.1013776)
Supplement: Attachment — Submitted filename: Response_to_Reviewers_auresp_2.docx [file ppat.1013776.s017.docx]

**Major Issues: Key Experiments Required for Acceptance**

**Reviewer #1:**

-------------------------------------------------------------------------------------------------------------------------

The authors have addressed most of the questions I raised in the revised manuscript and made the necessary revisions. However, I still have significant concerns about the design of the in vivo experiment in the final section. The authors' peptide has multiple antiviral objectives, yet only PBS was used as a control. This makes it impossible to discern whether the primary mechanism by which the peptide exerts its antiviral effects is through the cell surface entry mechanism discussed earlier or through some post-entry step. Furthermore, ASGR undergoes efficient endocytosis under ligand stimulation. If this is the case, the antiviral effects of the peptide would not logically align with the previously discussed mechanism of inhibiting viral entry.

-------------------------------------------------------------------------------------------------------------------------

We thank the reviewer for highlighting these critical issues regarding the peptide construct and the lack of an appropriate negative control. We fully agree that the multifunctional peptide design and the use of PBS alone as control limited the interpretability of the in vivo antiviral effects. In response to this concern, we restructured the in vivo section to focus exclusively on recombinant Netrin-1 (rNetrin-1) and removed all peptide-based experiments and figures from the manuscript. rNetrin-1 exhibited its antiviral activity on its own, without additional cargos, consistent with the cell-surface entry mechanism described earlier. Western blotting confirmed the localization of rNetrin-1 in liver tissue, indicating that the exogenously administered protein successfully reached the liver and suppressed HBV infection. However, the expected increase in serum Netrin-1 levels was not detected by ELISA. The follow-up pharmacokinetic analysis revealed that rNetrin-1 has a short systemic half-life in vivo, likely due to rapid turnover and efficient uptake by peripheral tissues, including the endothelium and liver. These properties may explain the unchanged serum Netrin-1 levels observed during administration.

We have revised the following text concerning the in vivo experiments: “Recombinant Netrin-1 inhibits HBV infection in humanized hepatocyte chimeric (TK-NOG) mice……………………. serum Netrin-1 reached a maximum concentration of approximately 800 pg/mL at 4 hours and subsequently declined to baseline levels within 24 hours (Supplemental Figure S13).” in the Results, pages 13-14, lines 422-446.

“Our in vivo studies demonstrate that exogenously administered rNetrin-1 successfully localized to liver tissue……………………. These pharmacokinetic properties likely account for the absence of detectable increases in serum Netrin-1 levels following exogenous rNetrin-1 administration in this study.” in the Discussion, pages 16-17, lines 518-540.

“Mice

TK-NOG mice were provided by the Central Institute for Experimental Animals (Kawasaki, Japan) ……………………. rNetrin-1 (5 µg in 100 µL PBS per dose, three times per week) was administered by intravenous injection via the tail vein.” in the Materials and Methods, page 24, lines 804–811.

We added the explanation of animal ethics as “All animal experiments were approved by the Ethics Committee for the Care and Use of Laboratory Animals at the Takara-Machi Campus of Kanazawa University………All experiments were performed in accordance with relevant guidelines and regulations.” in the Materials and Methods, page 24, lines 812–816.

“Recombinant human Netrin-1 purification

“Recombinant human Netrin-1 was expressed in HEK293F cells using the Expi293 Expression System Kit………..…. its purity was confirmed by SDS-PAGE followed by Coomassie Brilliant Blue staining and Western blotting.” in the Materials and Methods, pages 24-25, lines 818–834.

**Reviewer #2:**

-------------------------------------------------------------------------------------------------------------------------

1. Simplify and streamline the MS to focus on the likely mechanism of action of Netrin's anti-HBV effect.

2. Expand experimentally the part on the recombinant Netrin and its effect in vitro and in vivo using a proper control. Otherwise remove that section.

-------------------------------------------------------------------------------------------------------------------------

We thank the reviewer for the valuable suggestions. We fully agree that the multifunctional peptide, together with the lack of an appropriate negative control, limited the interpretability of the in vivo experiments and complicated the mechanistic conclusions. Following the reviewer’s recommendation, we newly evaluated the anti-HBV effect of purified recombinant Netrin-1 in vivo and removed the peptide data. The in vivo section now relies solely on rNetrin-1, which aligns with the entry-inhibition mechanism supported by our in vitro binding and transwell assays. rNetrin-1 alone demonstrated antiviral activity, consistent with the previously described cell-surface entry mechanism. Western blot analysis confirmed its accumulation in liver tissue, showing that the exogenously administered protein effectively reached the liver and inhibited HBV infection. Nevertheless, ELISA did not detect a corresponding increase in serum Netrin-1 levels. Follow-up pharmacokinetic studies indicated that rNetrin-1 has a short systemic half-life in vivo, likely due to rapid turnover and efficient uptake by peripheral tissues, including the endothelium and liver, which may explain the unchanged serum levels observed during administration. We have revised the Results and Discussion to present the in vivo findings. We believe this restructuring directly addresses the reviewer’s concerns and strengthens the mechanistic clarity of the manuscript.

We have revised the following text concerning the in vivo experiments: “Recombinant Netrin-1 inhibits HBV infection in humanized hepatocyte chimeric (TK-NOG) mice……………………. serum Netrin-1 reached a maximum concentration of approximately 800 pg/mL at 4 hours and subsequently declined to baseline levels within 24 hours (Supplemental Figure S13).” in the Results, pages 13-14, lines 422-446.

“Our in vivo studies demonstrate that exogenously administered rNetrin-1 successfully localized to liver tissue……………………. These pharmacokinetic properties likely account for the absence of detectable increases in serum Netrin-1 levels following exogenous rNetrin-1 administration in this study.” in the Discussion, pages 16-17, lines 518-540.

“Mice

TK-NOG mice were provided by the Central Institute for Experimental Animals (Kawasaki, Japan) ……………………. rNetrin-1 (5 µg in 100 µL PBS per dose, three times per week) was administered by intravenous injection via the tail vein.” in the Materials and Methods, page 24, lines 804–811.

We added the explanation of animal ethics as “All animal experiments were approved by the Ethics Committee for the Care and Use of Laboratory Animals at the Takara-Machi Campus of Kanazawa University………All experiments were performed in accordance with relevant guidelines and regulations.” in the Materials and Methods, page 24, lines 812–816.

“Recombinant human Netrin-1 purification

“Recombinant human Netrin-1 was expressed in HEK293F cells using the Expi293 Expression System Kit………..…. its purity was confirmed by SDS-PAGE followed by Coomassie Brilliant Blue staining and Western blotting.” in the Materials and Methods, pages 24-25, lines 818–834.

**Reviewer #3: No specific comment.**

**Minor Issues: Editorial and Data Presentation Modifications**

**Reviewer #1: No more comments.**

**Reviewer #2: (No Response)**

**Reviewer #3: No.**

We sincerely appreciate the reviewers’ insightful and constructive feedback. We have carefully addressed all comments and revised the manuscript accordingly. We hope that the updated version meets the requirements for publication in *PLOS Pathogens*.

Sincerely yours,

Masao Honda, MD, PhD

Department of Clinical Laboratory Medicine,

Kanazawa University Graduate School of Medical Science,

Kodatsuno 5-11-80, Kanazawa, 920-0942, Japan

+81-76-265-2601

Department of Gastroenterology,

Graduate School of Medicine,

Kanazawa University, Takara-Machi 13-1, Kanazawa 920-8641, Japan

Tel: +81-76-265-2235; Fax: +81-76-234-4250

E-mail: mhonda@m-kanazawa.jp
